# Experimental and Numerical Analysis of a Composite Thin-Walled Cylindrical Structures with Different Variants of Stiffeners, Subjected to Torsion

**DOI:** 10.3390/ma12193230

**Published:** 2019-10-02

**Authors:** Tomasz Kopecki, Przemysław Mazurek, Tomasz Lis

**Affiliations:** Faculty of Mechanical Engineering and Aeronautics, Rzeszów University of Technology, 35-959 Rzeszów, Poland; pmazurek@prz.edu.pl (P.M.); list@prz.edu.pl (T.L.)

**Keywords:** isogrid, aircraft load-bearing structures, finite elements method, nonlinear numerical analyses, stability, equilibrium path

## Abstract

The aim of the study was to determine the impact of the use of isogrid stiffeners on the stress and displacement distribution of a thin-walled cylindrical shell made of layered composites subjected to torsion. It also strives to define criteria for assessing the results of non-linear numerical analysis of models of the examined structures by comparing them with the results of the model experiment. The study contains the results of experimental research using models made of glass–epoxy composites and the results of numerical analyses in non-linear terms. The experiment was carried out using a special test stand. The research involved two types of considered structures. The results of the research allowed to create the concept of an adequate numerical model in terms of the finite element method, allowing to determine the distribution of stress and strain in the components of the studied structures. Simultaneously, the obtained conformity between the results of non-linear numerical analyses and the experiment allows to consider the results of analyses of the modified model in order to determine the properties of different stiffening variants as reliable. The presented research allows to determine the nature of the deformation of composite thin-walled structures in which local loss of stability of the covering is acceptable in the area of post-critical loads.

## 1. Introduction

Research devoted to the issue of loss of stability of systems that are elements of load-bearing structures used in technology, generally focuses on problems related to determining the value of critical loads. Analyses of post-critical conditions of structures become much more rarely the subjects thereof. This is due to the fact that in the vast majority of technical fields, the moment of loss of stability by the structure is identified with its destruction [1,2].

In aviation technology, due to the very specific nature of the objects under consideration, specific standards affecting design processes and operational assumptions have also been established. One of the principles, referring to the most commonly used in aviation metal structures, allows for post-critical deformations of selected types of systems, in the scope of operational loads [3,4].

In the general case, due to the need to minimize the mass of the object, loss of stability of the covering under operating conditions is allowed, if this phenomenon is elastic and occurs locally, i.e., within the shell segment limited by skeleton elements. The exceptions are coverings, e.g., of wing torsion box and other parts of the structure responsible for ensuring its appropriate torsional stiffness, as well as fragments of coverings, where large deformations are not desirable due to the need to maintain the aerodynamic properties [5,6].

Many years of research on aircraft structures, initiated by the Junkers construction office, have shown that limiting the area of post-critical deformations can be realized not only by increasing the number of skeleton elements. In many cases, an equally effective way to ensure the local nature of the phenomena has been the use of various forms of integral stiffeners. 

Although light metal systems are still the basic components of most of the aircraft load-bearing structures in operation, a clear tendency has emerged in recent years to increase the use of different types of composites. Layer composites are the most commonly used in aviation, based on glass, carbon and aramid fabrics, as well as polymeric resins [7].

Due to insufficient knowledge about the overall changes in the mechanical properties of composites caused by their long-term exploitation, the bearing structures based on them for many years were designed and implemented as shell-like, using the spacers to prevent loss of stability by bearing coverings. At present, in the pursuit of meeting increasingly strict operational and economic criteria, the design doctrine allowing the local loss of stability of some fragments of composite coverings is considered, e.g., in the case of metal. This type of assumption allows the use of semi-monocoque structures characterized by more favorable mechanical properties in relation to the mass than the layered monocoque structures [8,9].

The permissibility of loss of stability of composite coverings causes similar structural problems to appear as in the case of metal. One of them is the necessity to reduce this phenomenon, with as little weight increase as possible. Achieving this goal is possible through the use of stiffeners of an integral or "quasi-integral" nature.

**Figure 1:**
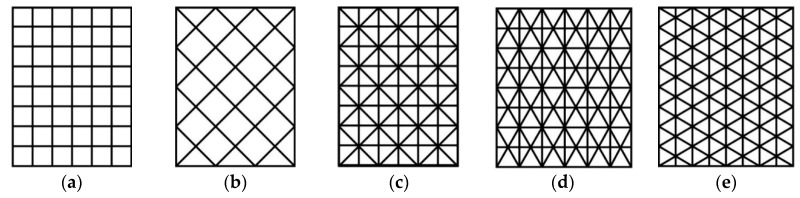
Schemes of grid stiffening structures: (**a**) ortho-grid, (**b**) x-grid, (**c**) bi-grid, (**d**) iso-grid type I, (**e**) iso-grid type II

The forms of integral stiffeners, which allow to obtain a significant increase in the stiffness of the covering, as well as relatively high values of critical loads, are grid structures (Figure 1). 

In the case of metal structures, the use of this type of solution is due to the need for precision machining, quite problematic (the need for precision machining). It is much simpler to realize it in the case of composite structures. As results from the published research results, the interest in constructors is focused mainly on isogrid structures [10,11]. Also, in the field of their applications, a number of experiments and numerical calculations have been made in composite constructions [12,13]. It should be emphasized, however, that in most cases the subject matter of the publication is limited to analyses of cylindrical shells subjected to compression. In the case of load-bearing systems used in aviation, the reason for the loss of stability of thin-walled systems is primarily torsion. This study focuses on the problem of local loss of stability of twisted shells, under the conditions of permissible loads and on post-buckling analysis.

## 2. Materials and Methods

**Figure 2:**
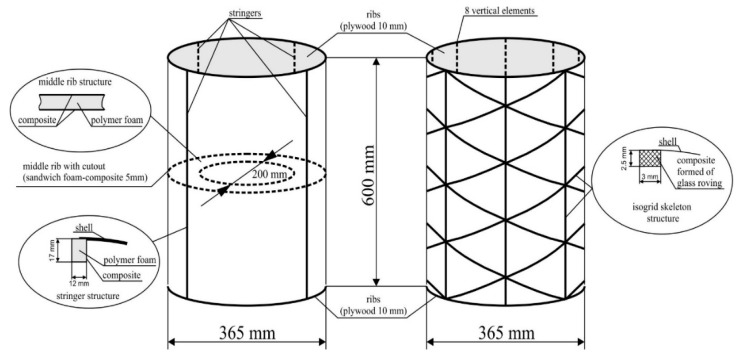
Schemes of models for experimental research: reference structure (on the left), structure with isogrid stiffener (on the right).

The aim of the research presented in this study was to perform a comparative analysis of two types of structural solutions of the aircraft structure fragment, represented by a thin-walled cylindrical structure with a composite covering, subject to post-critical deformations under operating conditions. The subject of the study were constitutions of equal dimensions (Figure 2), differing in structural solutions of the skeleton. The first of them was a reference structure with a stiffening corresponding to a classic semi-monocoque structure, consisting of four stringers and three ribs. External ribs were made of plywood; the mechanical properties are obtained with the constants defined in the aviation standards: E1 = 8500 MPa, E2 = 7500 MPa, G12 = 1000 MPa, ν12 = 0.34.

The second system had Type 2 isogrid stiffening. In both cases the shell of the model for experimental research was made as a composite structure which consisted of two layers of glass fabrics: 50 and 163 g/m^2^. Stringers of the reference structures were made as a closed circuit formed of two layers of glass fabric with a weight of 163 g/m^2^. The middle of every circuit was filled with polymer foam. The symmetric glass fabrics Interglass 02037 and 92110 were used to build the models. The matrix was a filling mixture based on epoxy resin MGS L285/H286 with known mechanical properties. Mechanical properties of the composite were obtained with the measured solid constants: E11 = 22,000 MPa, E22 = 22,000 MPa, ν12 = 0.11, G12 = 4600 MPa. Models were made using the contact method, with a 50/50 reinforcement ratio. The main directions of the composite orthotropy were oriented at 45 degrees to the direction of the axis of the cylindrical structure.

As it was proved during laboratory tests of some types of glass fabrics and epoxy resins applied in aviation, the physical constants for the single layer composites are almost identical in case of different fabrics with different weights [14]. So, the measured constants characterizes the behavior of each of layers.

The isogrid skeleton was formed by means of glass roving fibers in a polymeric mold, using the aforementioned filling mixture. 

The aim of the comparative analyses was to examine the differences between the character of post-critical deformations in both types of structures and the preliminary estimation of the impact of the form and size of deformations on the operational durability of the tested systems. 

The results of numerical analyses were evaluated by accepting the criterion of satisfactory similarity of the nature of post-critical deformations and representative equilibrium paths with reference to the results of the experiment. As a result, it became possible to determine stress distributions, based on the principle of unambiguity of solutions, according to which for an elastic system the deformation of the structure is responsible for one and only one variant of stress distribution.

**Figure 3:**
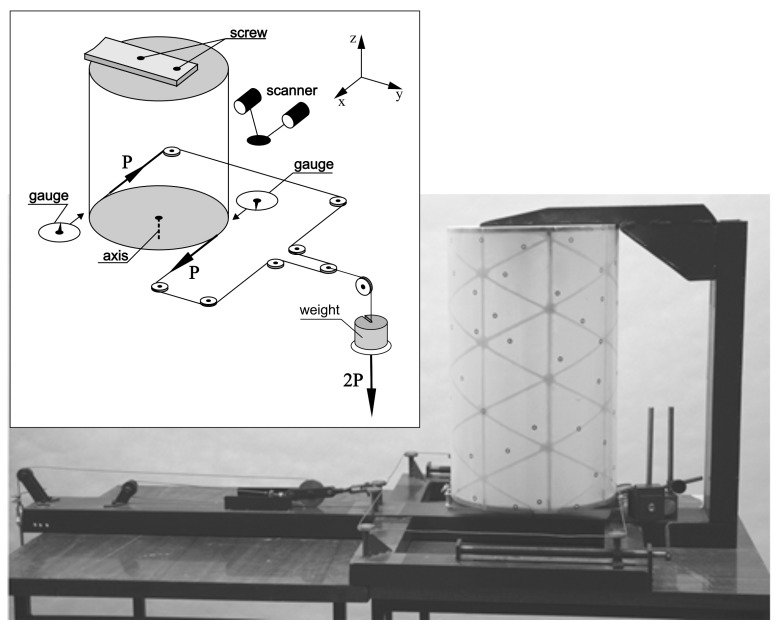
Stand for experimental research, load and deformation measurement diagram.

For the experiment, a special test stand was used, with high stiffness, whose own deformations can be considered negligibly small (Figure 3). Loads were carried out in a gravitational manner. The ATOS optical scanner and micrometer sensors were used for structure deformation measurements, on the basis of which the total torsion angle of the structure was determined. Due to the load application method, subsequent measurements were made for determined deformation states of the structure.

The results of experimental research constituted the material allowing us to obtain information about stress distributions in the tested systems by developing effective, adequate computational models in terms of the finite element method.

Numerical modelling of the analyzed structures was based on the commercial MSC PATRAN/MARC software (version 2012, MSC Software, Newport Beach, CA, USA), which proved its effectiveness in the case of post-critical deformations analyses of coverings made of isotropic materials [15]. In the case of layered composites, the key element of the software is an algorithm whose task is to determine the properties of the laminate, based on sets of constants corresponding to individual layers. In the case of commercial software used, this algorithm is an integral preprocessor procedure and does not allow the user to intervene.

The feature of composite structures which makes the creation of numerical mappings very difficult, is their heterogeneity, resulting not only from the conditions of lamination of individual layers, but also as a result of assembly operations, i.e., the presence of local surplus resin and varied thickness of glue joint. These factors may result in local changes in the stiffness of the covering and affect the form of post-critical deformations. Even small errors in the selection of geometric parameters of the numerical model, introducing a deviation from the actual boundary conditions of the shell segment, generate significant errors during non-linear analysis.

The basic relationship in a non-linear problem, defining the relationship between the state of the structure and the load is the so-called equilibrium path of the system, in general, a hypersurface in state hyperspace [16,17,18]. It is a relation that satisfies the matrix equation of residual forces:

(1) r(u,Λ)=0

in which u is a state vector, containing the displacement components of the structure nodes corresponding to its current geometric configuration, Λ is a matrix containing control parameters corresponding to the current load level, while r is a residual vector containing unbalanced force components related to the current state of system deformation.

In general, excluding the singularities resulting from the shape of the equilibrium path, it can be assumed that there are continuous relationships between the values contained in the above equation. For Clapeyron systems, the vector r for a fixed parameter value Λ is defined as a gradient of total potential energy Π (u,Λ) of the system:

(2) $$r=\frac{\partial \Pi }{\partial u}$$

which expresses that the condition of the static equilibrium of the system under consideration is a zero increase in potential energy.

Equation (1) can also be presented in the form of relationship:

(3) p(u)=f(u,Λ)

where **p** is a matrix containing internal forces corresponding to the current state of deformation, while f is a vector of external forces, which may also depend on the current state of deformation, which can be presented in the form of equations:

(4) $$p=\frac{\partial U}{\partial u},\;f=\frac{\partial P}{\partial u}$$

where **p** and **u** are respectively elastic strain energy and the work of external loads. The total potential energy of the system is expressed by the equation:

(5) Π=U−P

Stiffness matrix **K** of the system corresponding to the temporary, current configuration of the system is defined as a derivative of the residual vector r relative to the components of the state vector **u**:

(6) $$K=\frac{\partial r}{\partial u}$$

The **K**^−1^ inverse matrix is the system flexibility matrix. Excluding singularities corresponding to the characteristic points of the equilibrium path, both matrices are symmetric matrices.

By determining the derivative of the residual vector r relative to the control parameters, a control matrix, also called a load matrix, can be determined:

(7) $$Q=-\frac{\partial r}{\partial \Lambda }$$

The concept of stepwise changes in the configuration of the structure corresponding to the staged increase in load results in the possibility of binding the matrix **u** and **Λ** with a dimensionless parameter determining the degree of task completion, called the pseudo-time parameter:

(8) u=u(t), Λ=Λ(t)

The derivative of the residual vector component r in relation to the pseudo-time—**t** has the form:

(9) $$\overset{\circ }{r_{i}}=\frac{\partial r_{i}}{\partial u_{j}}\cdot \overset{\circ }{u_{j}}+\frac{\partial r_{i}}{\partial \Lambda_{i}}\cdot \overset{\circ }{\Lambda_{j}}$$

where:

(10) $$\overset{\circ }{r}=\frac{\partial r}{\partial t}$$

From the above compound and from dependences 6 and 7 the matrix equation follows:

(11) $$\overset{\circ }{r_{}}=K\cdot \overset{\circ }{u_{}}-Q\cdot \overset{\circ }{\Lambda_{}}$$

By determining the second derivative of the residual vector **r** relative to the pseudo-time parameter, we obtain:

(12) $$\overset{\circ \circ }{r}=K\cdot \overset{\circ \circ }{u}+\overset{\circ }{K}\cdot \overset{\circ }{u}-Q\cdot \overset{\circ \circ }{\Lambda }-Q\cdot \overset{\circ }{\Lambda }$$

where $\overset{\circ }{K}$ and $\overset{\circ }{Q}$ are matrices:

(13) $$\overset{\circ }{K}=\frac{\partial K}{\partial t},\overset{\circ }{Q}=\frac{\partial Q}{\partial t}$$

In numerical algorithms for non-linear problems, all components of the matrix is expressed as functions a single parameter *λ*, called the state control parameter. This parameter is a measure of the increase in the associated load, directly or indirectly, with the pseudo-time parameter—t. Thus, the equation of state 1 can be written in the form:

(14) r(u,λ)=0

called the monoparametric equation of residual forces. The corresponding derivatives in relation to the pseudo-time can be written as follows:

(15) $$\overset{\circ }{r}=K\cdot \overset{\circ }{u}-q\cdot \overset{\circ }{\lambda }$$

(16) $$\overset{\circ \circ }{r}=K\cdot \overset{\circ \circ }{u}+\overset{\circ }{K}\cdot \overset{\circ }{u}-q\cdot \overset{\circ \circ }{\lambda }-\overset{\circ }{q}\cdot \overset{\circ }{\lambda }$$

where $K=\frac{\partial r}{\partial u}$ is the defined by Equation (6) system stiffness matrix, also called the *tangent matrix* to the equilibrium path, while:

(17) $$q=-\frac{\partial r}{\partial \lambda }$$

is a *vector of load increase*.

Because at each stage of the solution a static equilibrium of the system is assumed, the vector r in each stage assumes zero values and does not change in relation to pseudo-time. The following relationships result:

(18) $$\overset{\circ }{r}=0\Rightarrow K\cdot \overset{\circ }{u}=q\cdot \overset{\circ }{\lambda }$$

(19) $$\overset{\circ \circ }{r}=0\Rightarrow K\cdot \overset{\circ \circ }{u}+\overset{\circ }{K}\cdot u=q\cdot \overset{\circ \circ }{\lambda }+\overset{\circ }{q}\cdot \overset{\circ }{\lambda }$$

Thus, for all points of the equilibrium path (for which K is a non-singular matrix), the relationship resulting from the Equation (18) can be used:

(20) $$\overset{\circ }{u}=K^{-1}\cdot q\cdot \overset{\circ }{\lambda }=v\cdot \overset{\circ }{\lambda }$$

where in

(21) $$v=K^{-1}\cdot q=\frac{\partial u}{\partial \lambda }$$

is a *vector of velocity of load increase*.

The prediction-correction methods of determining the consecutive points of the equilibrium path used in modern programs also include the correction phase based on the fulfillment by the system of an additional equation, called the increment control equation or the constraint Equation (18):

(22) $$c\left(\Delta u_{n},{\Delta \lambda }_{n}\right)=0$$

where the increases:

(23) $$\Delta u_{n}=u_{n+1}-u_{n}\;\mathrm{and}\;\Delta \lambda_{n}=\lambda_{n+1}-\lambda_{n}$$

correspond to the transition from state n to state n + 1.

As in the case of the experiment, since in the case of systems with the number of freedom greater than 2, it is difficult to interpret the equilibrium path in a clear graph form, in practice, for comparative purposes, representative equilibrium paths are used, which are the relationships between the chosen parameter characterizing deformation the system and a single control parameter related to the load. As a confirmation of the reliability of the results of non-linear numerical analyses in terms of FEM, it is considered that satisfactory convergence between representative equilibrium paths: determined during the experiment and obtained on the numerical way. It is also necessary to converge the forms of deformations that are the effects of calculations with the result of the experiment. Based on the aforementioned principle of unambiguity of solutions, the distributions of effective stress in the deformed shell can also be considered reliable. 

**Figure 4:**
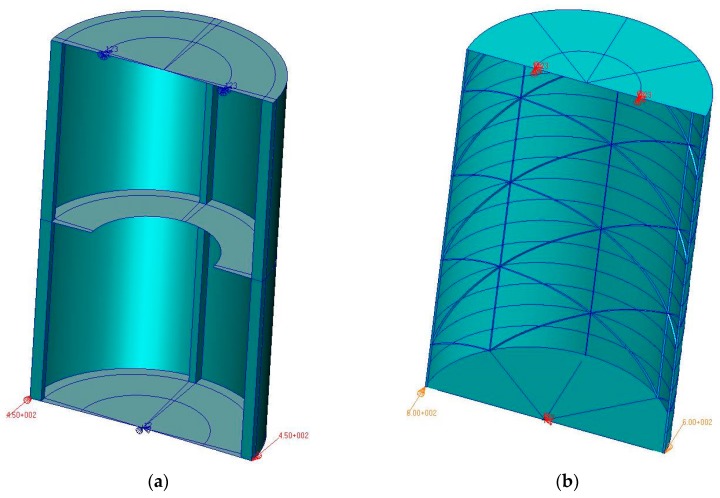
Fragments of geometric models, with visible methods of attachment and loads: (**a**) reference structure, (**b**) structure with isogrid.

The geometric structure of numerical models was based mainly on surface objects. In the case of the reference model, three-dimensional objects were also used, to model the stringers (Figure 4). It has to be emphasized that pictures below does not present complete models. Their present a half of each numerical model, for the better visualization of the structural details.

The use of this kind of solution resulted from the desire to map the actual proportions between the dimensions of the coverage segments.

The non-linear numerical analysis is an iterative process, aimed at determining subsequent equilibrium states, so its correctness is largely determined by the correct selection of the prediction method, correction strategy and a whole range of control parameters. In the described case, the Newton–Raphson method was used, related to the Crisfield hypersferrical correction [16,17].

After the series of numerical tests in the scope of choosing the topology of the model, the mesh consisting of about 3000 bilinear, four-node shell elements were used for the reference structure. The number of used elements was the result of analyses executed using various versions of the numerical models and it was a minimum providing the nonlinear analysis convergence and the compliance of results with the experiment. The necessity to a bilinear element resulted from the fact that other types of them, contained in the MSC MARC software library, which can be assigned to the properties of layered composites, do not have the ability to map geometrically complex objects, due to the type and number of degrees of freedom. 

For the modeling of the stringers, in the case of the reference structure, a total of 120 three-dimensional, eight-node elements were used. The structure model stiffened integrally was based entirely on surface elements, most of which were 4–node ones, the total number of which was 8700.

The material models were made taking into account the mechanical properties of composites based on the components used during the experimental phase, with the constants given above.

The process of nonlinear numerical calculations was multistage and a lot of mesh variants were tested. The goal of this procedure was to obtain the solution reproducing the results of experiment, however, under this procedure a quality of the mesh was also tested and analyzed. The convergence of the mesh was verified first, before the comparison with the experimental results were carried out, to obtain an assurance that in case of any mesh refinement the results do not change significantly. Presented and described results were obtained by means of mesh variants, which were considered as verified.

## 3. Results

### 3.1. Experimental Research

**Figure 5:**
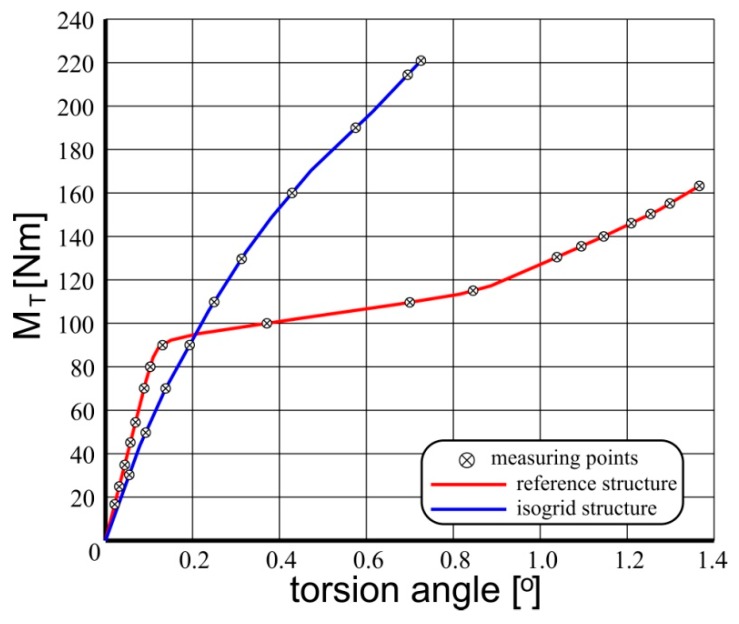
Representative equilibrium paths of the examined structures.

**Figure 6:**
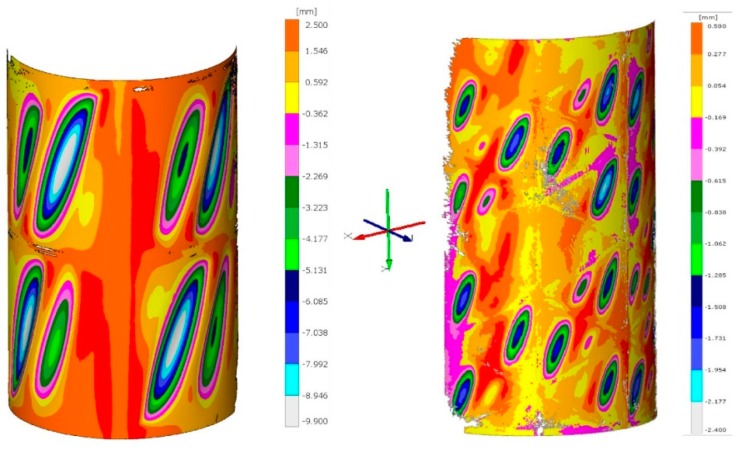
Distributions of deformations of the examined structures obtained as a result of scanning.

Measurements of total torsion angles of examined structures formed the basis for determining and comparing representative equilibrium paths of the studied systems (Figure 5). As the representative equilibrium path, the relationship between the said total torsion angle, characterizing the state of the structure and the control parameter for which the torsional moment was obtained, was assumed. For selected states, corresponding to the torsional moment values 160 Nm for the reference structure and 220 Nm for the structure with isogrid, the deformed surfaces of the structures were also scanned (Figure 6).

The shape of representative equilibrium paths indicates a completely different course of the phenomenon in both analyzed objects. In the case of a reference structure containing areas of coverage characterized by a high ratio of curvature to their surface area, the post-critical deformations occurred with pronounced skips, which was reflected in the occurrence of a linear section and refraction of the characteristic and a further significant increase in the torsion angle of the structure with relatively small increase of torsional moment. This corresponded to the emergence and deepening of large, double folds within each of the coverage segments. Although such deformations meet theoretical acceptability criteria, they cause a significant decrease in structural stiffness and deterioration of its operational properties. In the case of composite coverings, there is a serious concern that with such large deformations there may be local damage to the structure, e.g., in the form of delamination, resulting in a significant reduction in the operating durability of the system.

In the case of an integrally stiffened structure, the coverage segments between the frame elements constitute surfaces close to flat ones, as a result of which a loss of stability within the majority of them occurs at a relatively low load value. However, the structure quickly achieves a state of post-critical equilibrium, gaining high stiffness, thanks to which a representative equilibrium path maintains a linear-like character even with high load values. The depth of folds formed within the coverage segments is small, which allows to conclude that the structure has a much higher durability compared to the reference system.

It should also be emphasized that the masses of both structures are very similar (1849 g, the reference structure, 1861 g, the structure stiffened integrally), while the stiffness of the structure with the isogrid type is almost 70% higher than the stiffness of the reference structure.

### 3.2. Nonlinear Numerical Analyzes

**Figure 7:**
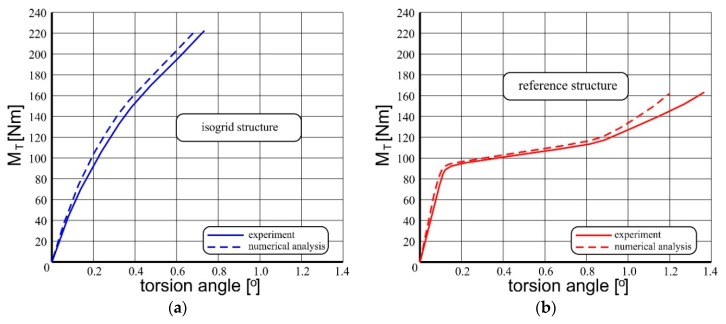
List of representative equilibrium paths obtained in numerical way and as a result of the experiment: (**a**) structure stiffened with isogrid, (**b**) reference structure.

As a result of conducted non-linear numerical analyses, representative equilibrium paths were determined and their comparison was made with the appropriate characteristics obtained during the experiment (Figure 7).

In both cases, the stiffens of the numerical models were slightly higher stiff then their experimental counterparts. It results from the simplifications, which must be applied due to limited effectiveness and efficiency of the numerical procedures applied in commercial FEM software. 

**Figure 8:**
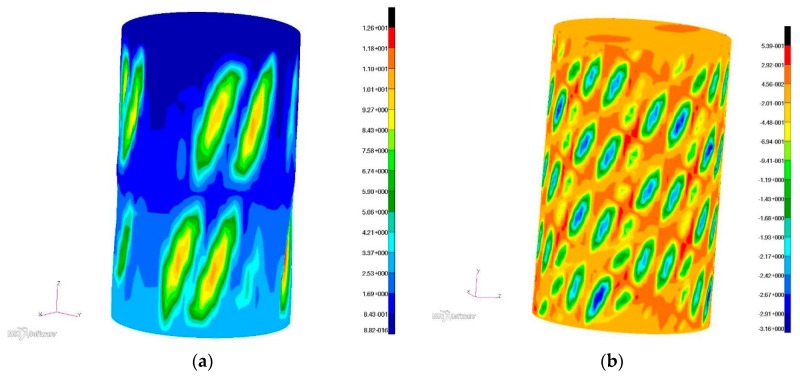
Distributions of the radial deformation component at the maximum load value: (**a**) reference structure, (**b**) structure with isogrid.

Both numerical models were characterized by slightly higher stiffness than experimental models, although the deformation of some parts of the covering in both cases turned out to be greater than in the case of the latter (Figure 8). This is a consequence of the fact that the state of the structure depends on the proportion between many parameters defining it. As a result, different combinations of state parameters in different subsets may result in a similar value of a representative parameter which in the described case, is the total torsion angle.

However, despite these divergences, the results of non-linear numerical analyzes can be considered satisfactory. In particular, in the case of the isogrid structure, the inconsistency of the representative equilibrium paths did not exceed 7%. On the other hand, in the case of the reference structure, this inconsistency for most of the range of post-critical deformations did not exceed 15%.

**Figure 9:**
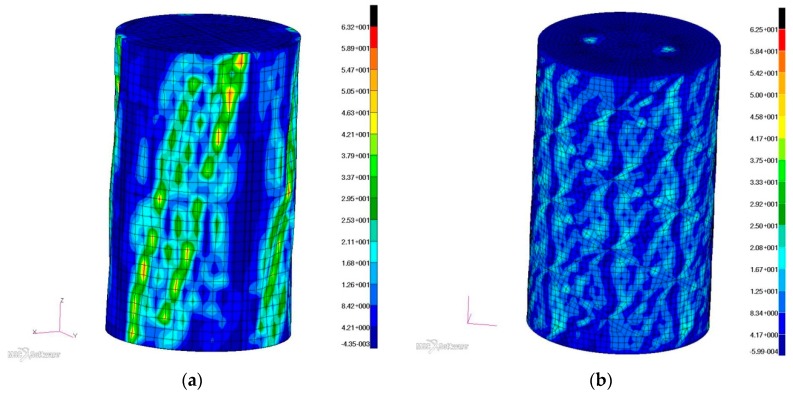
Distribution of effective stress according to the hypothesis of the highest tensile stress: (**a**) reference structure, (**b**) structure with isogrid.

Satisfactory similarity of the form of post-critical deformations was also achieved, and based on the above-mentioned principle of unambiguity of solutions, the distribution of effective stress based on the hypothesis of the highest tensile stress can be considered reliable (Figure 9). However, stress levels in the cylindrical part of the structure stiffened with isogrid were much lower than in the reference structure, for better visual comparison of the two cases of stress distribution, similar maximum scales were applied.

## 4. Discussion

The analysis of the nature of post-critical deformations of the considered systems and the comparison of their masses, reveals a number of advantages of a structural solution based on an isogrid integral stiffener. The semi-monocoque reference structure used for the study was an example of the use of a minimum number of skeleton elements, as a result of which relatively large covering segments with significant curvature underwent deformation. In this case, the nature of the deformation may cause a significant reduction in the reliability of the structure, resulting from the possibility of local damage of the composite. Traditional solutions used in aviation technology rely on increasing the number of frames and stringers. Due to such a solution the covering segments gain smaller dimensions, and the relations between them and the shell curvature become more advantageous. However, increasing the number of skeleton elements leads to a significant increase in the mass of the structure. Therefore, if additional frames or stringers are used, the semi-monocoque structure would have to have a larger mass than the structure with isogrid type stiffeners.

It should be emphasized that inference about the operational lifetime of a structure, regardless of the material used, requires knowledge about its behavior under cyclic loading conditions. However, even in the absence of such information, in the case of static studies, it can be assumed that local damage to the structure may appear primarily in the areas of high stress gradients. From this point of view, the structure with isogrid type stiffeners has a great advantage, characterized by a very regular distribution of stresses that is devoid of explicit concentrations. 

The results of experimental studies allowed the development of adequate numerical models based on the finite element method, using commercial software. This allows to conclude that the processes of designing thin-walled composite structures subjected to post-critical deformations under the conditions of permissible loads can be numerically assisted, however the results of numerical analyses should be verified experimentally, even using a simplified model experiment. The numerical models presented in this study were subjected to repeated tests and in a number of them, erroneous results were obtained, characterized by incompatibility with the actual deformations and the deformation of the analysed systems. Relatively simple and cheap experiment allowed to correct errors and selection of the most appropriate sets of numerical procedures, as well as parameters controlling the course of computational processes. This confirms the validity of the thesis that relying on unverified results of numerical analyses may lead to the appearance of significant construction errors.

## 5. Conclusions

The presented study was limited to the analysis of one type of isogrid stiffening, due to the inconvenient and time-consuming process of creating a model for experimental research. The search for the most appropriate solution, from the point of view of distribution of stresses and strains, would have to include the analysis of a whole range of stiffening variants and their impact on the aforementioned size and total mass of the structure. In this context, the methodology of gradual modifications of the numerical model verified by the experiment, aimed at testing successive versions of structural solutions, seems to be useful. However, it should be emphasized that the greater the deviation of the numerical model from its verified form, the greater the probability of obtaining incorrect results. Therefore, the final stage of the analysis seems to be the final experimental verification of the solution considered to be the most effective.

The presented results should be evaluated in the context of the expected broader scope of research, aimed at determining the best possible proportions between the dimensions of the structure and the number and form of integral stiffening elements. It should be emphasized that it is also planned to subject selected representative systems to cyclic loads and to attempt to develop an effective method of detecting emerging damages.
